# Adult Stem Cell-Derived Extracellular Vesicles in Cancer Treatment: Opportunities and Challenges

**DOI:** 10.3390/cells9051171

**Published:** 2020-05-08

**Authors:** Vadims Parfejevs, Krizia Sagini, Arturs Buss, Kristine Sobolevska, Alicia Llorente, Una Riekstina, Arturs Abols

**Affiliations:** 1 Faculty of Medicine, University of Latvia, House of Science, Jelgavas Str 3, LV-1004 Riga, Latvia; vadims.parfejevs@lu.lv (V.P.); una.riekstina@lu.lv (U.R.); 2 Department of Molecular Cell Biology, Institute for Cancer Research, Oslo University Hospital, 0379 Oslo, Norway; Krizia.Sagini@rr-research.no (K.S.); alicia.llorente@ous-research.no (A.L.); 3 Latvian Biomedical Research and Study Centre, Ratsupites Str 1, k-1, LV-1067 Riga, Latvia; arturs.buss@biomed.lu.lv (A.B.); k.sobolevska@gmail.com (K.S.)

**Keywords:** extracellular vesicles, mesenchymal stromal cells, cancer treatment

## Abstract

Adult stem cells (SCs) participate in tissue repair and homeostasis regulation. The relative ease of SC handling and their therapeutic effect has made of these cell popular candidates for cellular therapy. However, several problems interfere with their clinical application in cancer treatment, like safety issues, unpredictable pro-tumour effects, and tissue entrapment. Therefore cell-free therapies that exhibit SC properties are being investigated. It is now well known that adult SCs exhibit their therapeutic effect via paracrine mechanisms. In addition to secretory proteins, SCs also release extracellular vesicles (EV) that deliver their contents to the target cells. Cancer treatment is one of the most promising applications of SC-EVs. Moreover, SC-EVs could be modified to improve targeted drug delivery. The aim of the review is to summarise current knowledge of adult SC-EV application in cancer treatment and to emphasise future opportunities and challenges in cancer treatment.

## 1. Introduction

Adult mammalian stem cells (SCs) are found in various organs, where they reside in specific niche environments and participate in tissue homeostasis [1]. However, it appears that many human organs lack cells that meet stringent SC criteria, and a more open research approach focusing on the function of SCs instead of their physical entity may be useful [2]. Mesenchymal stromal cells (MSCs) as transplantable repair cells could fit into such broader criteria. Their SC function should then be well demonstrated, similarly to the function of bone marrow MSCs (BM-MSCs) in bone marrow niche formation [3,4].

MSCs are a culture-adapted heterogeneous population of cells that can be established from various tissues, most notably from the bone marrow (BM-MSCs), fat (AD-MSCs), and umbilical cord (UC-MSCs). MSCs are identified based on a set of surface markers and differentiation towards adipocyte, chondrocyte, and osteocyte phenotypes in vitro [5]. It is a bulk population of cells representing a mix of various clones of committed progenitors that can include SCs from the original tissue [6]. Despite the common name, which is a subject of debate [7], MSCs from different organs display distinct gene expression patterns, DNA methylation status, and in vivo differentiation potential [8,9]. The precise location and function of endogenous MSCs equivalents are also elusive, but the current data suggest that the origin of MSCs in various tissues is the perivascular niche [10,11]. Such an association with the vasculature would make MSCs omnipresent, and it is tempting to assume that these cells are ready to respond to injury and contribute to local tissue repair [9]. However, genetic tracing experiments have lately put a shadow on this assumption and suggest that there may be other explanation for the origin of MSCs [12,13]. Whether endogenous MSCs can be mobilised from distant locations and home to the sites of injury remains controversial [14]. However, the migration of transplanted MSCs towards tumours and injury sites has been demonstrated [15].

The relative ease of MSC handling and clinical safety have made of them popular candidates for cellular therapy [16]. A search for ‘mesenchymal stem cells’ on ClinicalTrials.gov returns more than 1000 results. The therapeutic potential of MSCs in various malignancies is the subject of many clinical trials [17]. Vast pre-clinical data suggest the involvement of these cells in virtually all important aspects of cancer progression including tumour growth [18], epithelial to mesenchymal transition (EMT) [19,20], the formation of the tumour microenvironment [21], angiogenesis [22], immune response [23], and drug resistance [24]. Both pro- and anti-cancer effects of MSCs have been observed in these studies. For extensive reviews on the topic, see references [17,25]. Promising data from animal studies are, unfortunately, often followed with underwhelming performance in clinical settings [16]. Nevertheless, clinical trials have demonstrated the beneficial effects of MSCs in conditions like steroid-refractory graft versus host disease [26] and complications of Crohn’s disease [27]. This has resulted in MSC-based products reaching the market (e.g., Alofisel^TM^), and some adjustments like augmenting the properties of MSCs, finding the right therapeutic window, and improving the clinical trial design, might bring more success in the future [16,28].

The mode of action of MSCs is not completely understood but is likely associated with soluble factors and extracellular vesicles (EVs) derived from these cells acting in a paracrine manner to achieve immunomodulatory and pro-regenerative effects [29]. In fact, MSC-derived EVs are considered as an alternative to MSCs in a cell-free therapeutic approach for various clinical indications [30]. Therefore, in this review, we will focus on EVs derived from adult SCs, and specifically on the most investigated ones, the MSCs.

EVs are a heterogeneous group of membrane-enclosed vesicles that are released by various types of cells, including SCs [31]. Initially, EVs were considered as cell debris in the 1940s [32], but now they have become one of the most widely studied means of intercellular communication [33]. Their ability to transfer different types of molecules from cell to cell [34] and influence the behavior of recipient cells has led to an increased amount of studies about their role in cancer progression and potential applications in cancer treatment [35,36,37,38].

Due to their highly heterogeneous nature and overlapping size and markers, it is challenging to categorise EVs into subtypes [39]. Nevertheless, based on their biogenesis EVs are generally classified into three groups: exosomes, microvesicles (MVs), and apoptotic bodies [31]. Exosomes usually have a diameter of 30 to 150 nm and are capable of passing through biological barriers like the blood-brain barrier (BBB) [40]. They originate in multivesicular bodies (MVBs) and are secreted into the extracellular space upon MVB fusion with the plasma membrane [41]. MVs, often termed ectosomes or microparticles, are vesicles of 100 to 1000 nm in diameter that are formed by outward budding of the plasma membrane followed by a fusion step [42,43]. Apoptotic bodies, which are released as blebs from apoptotic cells, are usually larger EVs ranging in diameter from 1 to 5 μm [44]. Although EV biogenesis routes have been studied to some degree, the molecular machinery of these processes is not completely understood. For extensive reviews of EV classification, biogenesis, uptake, and functions, see [33,45,46,47,48]. In the current review, we will use the ISEV (International Society of Extracellular Vesicles) accepted generic term, EVs, which covers all particles naturally released from the cell that are delimited by a lipid bilayer and cannot replicate [39].

This review focuses on the current state-of-the-art applications of adult SC-derived EVs in cancer. We will emphasise future implications, challenges, and opportunities these EVs hold for cancer treatment and highlight their progress into the clinic. 

## 2. Stem Cell-Derived EVs

**Figure 1:**
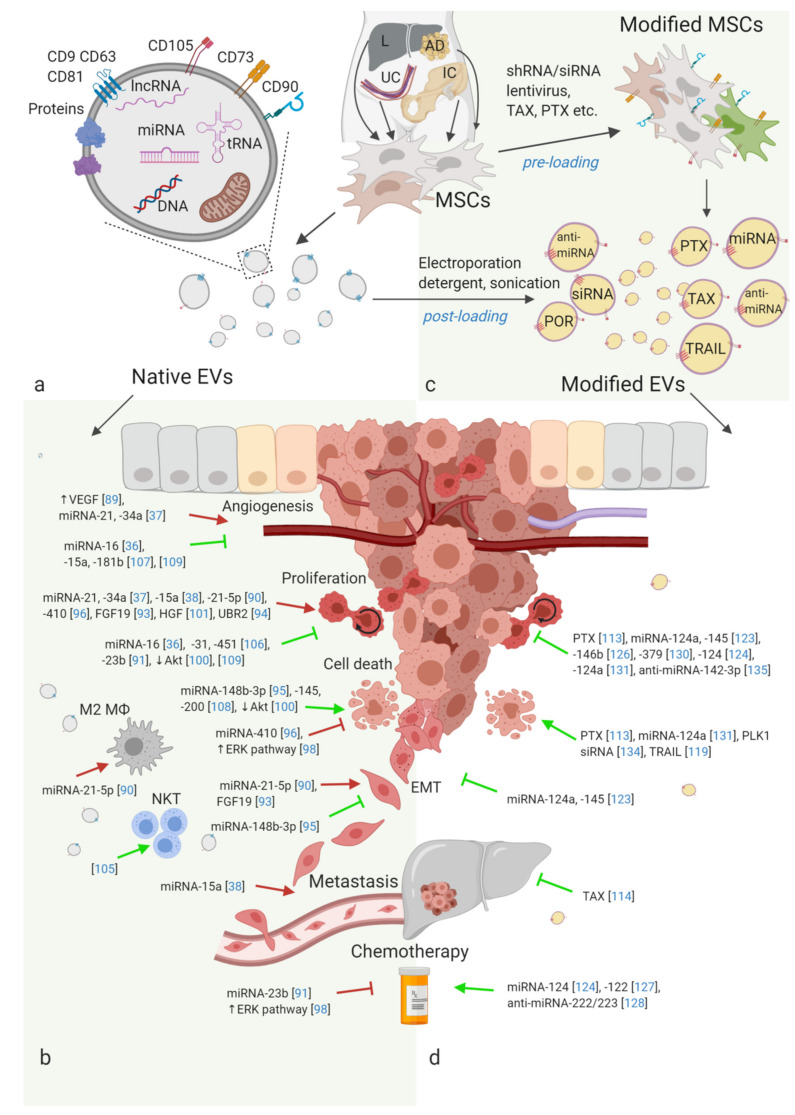
Inhibitory and tumour promoting effects of native and modified EVs from adult SC populations in preclinical in vivo cancer models. (**a**) MSCs can be derived from various adult human tissues, e.g., bone marrow of the iliac crest, adipose tissue, umbilical cord, liver, and many others. Unmodified MSCs are a source of native EVs that can carry a rich cargo of various nucleic acids, lipids, proteins, and even organelles like mitochondria. (**b**) Native SC-EV inhibitory and tumour promoting action. Key publications and molecules implicated in the observed effect on important processes of tumour progression, including angiogenesis, proliferation, cell death, EMT, immune evasion, metastasis, and resistance to chemotherapy. (**c)** SC-EVs can be modified in many different ways. Pre-loading methods imply altering the cell to modify EV cargo. Post-loading methods aim to package the desired molecules inside of the isolated EVs. Here we mention the methods discussed in this review. (**d**) Modified SC-EV effects in tumour xenograft models. The most important in vivo findings are highlighted. Abbreviations: L—liver, UC—umbilical cord, IC—iliac crest, AD—adipose tissue, TAX—taxol, PAX—paclitaxel, POR—porphyrins, shRNA—small hairpin RNA, siRNA—small interfering RNA, lncRNA—long non-coding RNA, tRNA—transport RNA, NKT—natural killer T cell, M2 MФ—M2 polarised macrophage. Image created using BioRender.com.

It has been shown that EVs are released by progenitor cells like MSCs [49,50], endothelial progenitor cells (EPCs) [51], neural SCs (NSCs) [52], induced pluripotent SCs (iPSC) [53,54], embryonic SCs (ESCs) [55,56], and cardiomyocyte progenitor cells (CPCs) [57]. SC-derived EVs, similar to EVs released by other cell types, contain signaling proteins, ECM proteins, growth factors, transcription factors, metabolic enzymes, DNA and RNA-binding proteins, as well as different types of RNA and both genomic and mitochondrial DNA (Figure 1a) [39,58].

While it is well recognised that the content of EVs is dependent on their biogenesis pathway, the cell type of origin, and physiological conditions [39], it also has become clear that SC-EV release, cargo, and functions are influenced by the surrounding microenvironment [59,60]. For example, Lai et al. performed proteome analyses of EVs released at three time points from MSCs and detected 379, 432, and 420 proteins [61]. Only 154 proteins were found in all three samples. Such dynamic changes in EV content are currently one of the reasons that hamper research progress in the field.

In an attempt to standardise the methodology, a recent position paper suggested that MSCs-EV characterisation should follow the “minimal Information for studies of EVs” (MISEV2018) guidelines [39], and minimal criteria defining MSCs immunophenotype should be attributed to MSCs-EV characterisation. Ramos et al. have neatly demonstrated that, in addition to EV-specific markers such as CD63, CD9, and CD81, MSC-EVs bear MSC markers such as CD29, CD73, CD90, CD44, and CD105 (Figure 1a) [62]. A meta-analysis of the MSCs-EV proteome revealed an enrichment of cell- and protein-adhesion proteins, and particularly collagens I and VI were abundant in MSC-EVs [63]. Also, several studies show that MSC-EVs contain high levels of interleukins (IL) IL-6, IL-8, IL-10, and other cytokines [61,64,65]. Meanwhile, analysis of BM-MSC- and UC-MSC-derived EVs revealed a presence of different immunomodulatory protein coding mRNAs and different anti-inflammatory and tissue healing potentials in target cells [66]. Similarly, MSC-EV enclosed miRNAs also contribute to regeneration, inflammation, and angiogenesis through various related signaling pathways [67]. A comparative high-resolution lipidomic analysis of large and small EVs derived from BM-MSCs, glioblastoma (U87), and hepatocellular carcinoma (Huh7) cells showed that acylcarnitines and lysophosphatidylcholines where enriched only in large MSC-EVs. Moreover, lysoderivatives of phosphatidylserines, phosphatidylglycerols, and phosphatidylinositols showed enrichment in small MSC- and Huh7-EVs. These lysoderivatives were also enriched in large MSC-EVs, but depleted from large U87- and Huh7-EVs. Taken together, these results indicate that MSCs release large and small EVs with a unique lipid composition compared to other cells [68]. Next, EV membrane protein studies suggest that adult SC-EVs contain certain membrane proteins, like prominin-1 (also known as CD133) and prominin-2 that bind to specific lipids, such as cholesterol [69,70]. Larger cargo, like whole organelles, can also be shuttled to other cells by EVs. MSCs experiencing oxidative stress, for example, can dispose of depolarised mitochondria and transfer them to macrophages [71]. Such fueling of neighboring macrophages via EVs represents an intricate way by which MSCs can perform immunomodulation.

Equipped with such a broad range of active signaling molecules, SC-EVs, as mediators of cell-to-cell communication, seem to participate in many fundamental biological processes like development [55] and tissue homeostasis [72,73]. Numerous studies have highlighted the role of progenitor/SC -derived EVs in tissue repair. MSC-EVs, for example, have been reported to promote wound healing [74], enhance the hepatic regeneration after liver damage [75], protect against hypoxia-induced lung injuries [76] and lessen kidney trauma [77]. Furthermore, EPC-EVs have been reported to promote neovascularisation in hindlimb ischemia in vivo [78], and CPC-EVs are suggested to improve cardiac functions after myocardial injury [79,80]. Interestingly, iPSC-EVs are more effective for cardiac repair than iPSCs [54]. Additionally, iPSC-EVs may also reduce liver fibrosis in vivo [53]. Altogether, these studies support the therapeutical potential of SC-EVs.

Many hallmark processes of cancer progression such as increased proliferation, cell migration, angiogenesis, inflammation, and extracellular matrix (ECM) remodeling have parallels with the events seen in wound repair and fibrosis [81] to the extent that tumours are viewed as wounds that fail to heal [82,83]. SC-EVs seem to play a dual role in cancer and can either promote or suppress cancer progression. For example, BM-MSC and UC-MSC derived EVs decrease proliferation and induce apoptosis of glioblastoma cells, whereas AD-MSC-EVs enhanced tumour cell growth and had no effect on apoptosis [35].

These contradicting observations suggest that adult SCs-EV functions can be highly dependent on their cell of origin and their cargo. The involvement of normal endogenous adult SCs in cancer progression is not well understood, apart from observations that cancer can develop as a result of mutations in adult SCs, for example, in the intestine [84]. Even less is known about the endogenous SCs-derived EVs and their role in cancer. There is some evidence for EV-mediated interaction between SCs and other cells in the SC niche, e.g., in the way a crucial SCs factor such as Wnt is transferred [85], but the significance of such EVs for tissue regeneration or cancer in vivo remains to be further elucidated [86].

Building on the knowledge of SC-EVs, different studies are exploring the potential of these EVs as carriers for drug delivery in cancer therapy. Multiple approaches have been described to improve the selectivity, efficiency, and safety of therapeutic EVs. The strategies for using SC-EVs in cancer treatment can be divided into two major categories: (i) using native MSC-derived EVs, and (ii) modification of SC-EVs by targeting parent SCs (pre-loading) or targeting EVs after their release (post-loading) [87,88]. The role of native and modified EVs will be discussed in the following sections.

## 3. Unmodified (Native) Adult SCs-EVs Effect on Cancer

In this chapter, we will give a comprehensive summary of research performed with native SC-EVs in various cancer models. Most of the studies have investigated the effects in vitro and have been followed up by xenograft models. The studies included in this review are summarised in Table 1 and Table 2 and Figure 1b.

### 3.1. EVs from BM-MSCs

Many studies have investigated BM-MSC-derived EVs. An early report showed that these EVs decreased cell proliferation in several cancer cell lines (HEPG2 hepatoma, Kaposi sarcoma, SK-OV-3 ovarian) in vitro, as well as induced cell cycle arrest and reduced tumour burden in xenograft models [50]. Likewise, Lee at al. found a reduced proliferation in the breast cancer cell line 4T1 and observed that BM-MSC-EVs down-regulate vascular endothelial growth factor (VEGF) expression in tumour cells, which results in the inhibition of angiogenesis both in vitro and in a subcutaneous xenograft [36]. These effects were partially attributed to miRNA-16 present in BM-MSC-EVs, which down-regulates VEGF in 4T1 cells.

A thorough study addressed the importance of the source of human BM-MSCs for the generation of EVs. Whereas multiple myeloma (MM) patient-derived BM-MSC-EVs promoted tumour growth and homing to the bone marrow, normal MSC-EVs inhibited the growth of MM cells and metastasis formation [38]. A study of the molecular composition of MSC-EVs derived from MM patients found enrichment in oncogenic proteins, cytokines, and adhesion molecules, and lower levels of the tumour suppressor miRNA-15a compared to MSC-EVs from healthy donors. This led the authors to hypothesise that BM-MSCs use EVs to modulate tumour cells and form a supportive niche in MM.

Zhu and colleagues reported that human BM-MSC-EVs can promote tumour growth [89]. Contrary to observations in breast cancer cells [36], in this study, EVs promoted VEGF expression in gastric carcinoma SGC-7901 and colon cancer SW480 cells, prompting angiogenesis and xenograft growth [89]. BM-MSC-EVs exhibited tumour supportive effects in another breast cancer line (MCF-7) [37]. Vallabhaneni and colleagues investigated the composition of these EVs and identified a range of potentially tumour-supportive proteins. The authors also noted that stressful culture conditions influence the composition of MSC-EVs. In fact, serum-deprived MSCs release EVs containing higher levels of tumour-promoting miRNAs, such as miRNA-21 and miRNA-34a, that favour breast cancer cell proliferation and metastasis in a xenograft model [37]. Moreover, Ren et al. reported that EVs secreted by BM-MSCs under hypoxia increased A549 and H23 (lung cancer) cell proliferation, survival, invasiveness, and EMT as well as decreased apoptosis and macrophage M2 polarisation in a xenograft model by increasing the delivery of miRNA-21-5p [91]. Another study demonstrated that the co-culture of the metastatic breast cancer cell line MDA-MB-231 with BM-MSC-EVs resulted in decreased sensitivity to docetaxel and acquisition of the dormancy features in breast cancer cells. In subsequent experiments, such effect was attributed to miRNA-23b enclosed in EVs, which can target the expression of MARCKS, a protein known to promote proliferation and cell motility [92].

In addition, Qi et al. showed that BM-MSC-EVs can promote the growth of MG63 osteosarcoma cells and SGC7901 gastric cancer cells through activation of hedgehog (HH) signalling in vitro [90], while the EV-activated FGF19-FGFR4-ERK signalling axis has been implicated in nasopharyngeal carcinoma xenograft formation and growth [93]. The authors suggested that increased FGF19 protein content in EVs stimulates EMT and migration of tumour cells [93]. Another interesting finding was reported by Mao and colleagues, who showed that p53-/- knockout mouse primary BM-MSCs produce more EVs, which are enriched in UBR2. Treatment of p53+/+ primary mouse BM-MSCs and MFC (murine foregastric carcinoma) with these EVs resulted in overexpression of UBR2 in target cells and elevated cell proliferation, migration, and expression of stemness related genes [94].

### 3.2. EVs from UC-MSCs

Similarly, both pro- and anti-tumour effects are observed using human UC-MSC-EVs. For example, UC-MSC-EVs carrying miRNA-148-3p were recently shown to regulate the expression of tripartite motif 59 (TRIM59), a protein involved in malignancy and overexpressed in some cancer types. In MDA-MB-231 breast cancer cell xenografts, these EVs were linked to stimulated apoptosis, decreased proliferation, and diminished levels of EMT-related proteins [115].

The opposite effects, namely increased proliferation and apoptosis prevention, were observed for UC-MSC-EVs in a lung adenocarcinoma model. Pre-treatment of cancer cells with UC-MSC-EVs markedly increased the xenograft size by transferring miRNA-410 to tumour cells and causing a decrease in PTEN expression [98]. Another study suggested an EV-mediated transfer of proteins (matrix metalloproteinase 2 and 5’-nucleotidase) and their enzymatic activity to MCF-7 breast cancer cells and SCCOHT-1 ovarian cancer cells, and subsequent increase in cancer cell heterogeneity [99]. Along these lines, UC-MSC-EVs were shown to activate calmodulin kinase Raf/MEK/ERK signaling axis in several gastric cancer cell lines, and partially reverse the anti-tumor effects of 5-fluorouracil in a xenograft model [97]. This is another demonstration of MSC-EVs being able to confer resistance to chemotherapy. Such effects can also be seen as protective, e.g., UC-MSC-EVs can prevent cisplatin-induced apoptosis of ovarian granulosa cells, which is correlated to primary ovarian insufficiency and chemotherapy-related infertility [125].

Not surprisingly, EVs derived from Wharton’s jelly MSCs (WJ-MSCs) of the umbilical cord also demonstrated contrasting effects in different models. In one study, the growth of a T24 bladder carcinoma cell xenograft was inhibited by WJ-MSC-EVs through the downregulation of the Akt pathway and activation of cleaved Caspase 3 [116]. However, the incidence and growth of tumours from xenografts of renal cell carcinoma cells were increased and associated with higher hepatocyte growth factor (HGF) expression in the tumour cells [100].

### 3.3. EVs from AD-MSCs

So far, only a few studies have tested the effects of EVs from AD-MSCs. Lin et al. demonstrated that human AD-MSC-EVs can contribute to tumour cell migration in vitro [96]. Other in vitro studies found tumour inhibitory effects. It has been shown that AD-MSC-EVs decreased the proliferation of the ovarian cancer cell lines SK-OV-3 and A2780 and hampered their migration ability and clonogenicity [112]. Another group showed that AD-MSC-EVs carry miRNA-145 that potentially inhibits the growth of prostate cancer cells (PC3M). The knockdown of this miRNA abrogated the anti-proliferative and pro-apoptotic effects of AD-MSC-EVs [111].

EVs derived from rat AD-MSCs were studied in an N1S1 cell line induced rat hepatocellular carcinoma. The animal group that received systemic EV administration displayed improved tumour grading, while histological and blood sample analysis revealed an increase in the numbers of circulating and intratumoural natural killer T-cells in these animals. The results from this orthotopic tumour cell transplantation model provided a rare link to a potential immunomodulatory effect of MSC-EVs [113]. 

### 3.4. EVs From Other Stem/Progenitor Cells

Several studies have looked at the properties of EVs derived from MSC-like cells. An early study found the growth of an HepG2 hepatoma xenograft to be reduced in animals treated with liver MSC-EVs, and this effect was correlated with the delivery of miRNA-451 and miRNA-31 to the tumour cells [119]. A later study by Lopatina and colleagues demonstrated that liver MSC-EVs can inhibit the angiogenesis potential of tumour-derived endothelial cells in vivo [120]. Furthermore, in contrast to EVs from the liver, BM-MSC-EVs were found to be highly angiogenic. Authors suggested that several nucleic acid molecules could be involved, including miRNA-15a, -181b, -320c, and -874. Moreover, it was recently tested if the liver MSC-EVs could inhibit renal cancer stem cell (CSC) growth [121]. Pre-treatment of renal cancer cells with EVs from both liver and BM MSCs resulted in delayed tumour development in immunocompromised mice. Corroborating the previous observations, intravenous treatment with EVs from liver MSCs and not BM-MSCs impaired xenograft growth and vascularisation and resulted in improved survival. The tumour-suppressive effects are possibly tied to the transfer of miRNA-145 and miRNA-200 [121]. A hamster model of chemically-induced oral squamous cell carcinoma (OSCC) was used to study the effect of EVs derived from human menstrual fluid MSCs (MenSC). EVs were repeatedly injected at the base of the tumour, which markedly slowed further growth of the neoplasia and reduced the density and area of the vasculature [124].

The studies listed above signify the inconsistency of native SC-EV action in tumour models. The opposite effects of native SC-EVs in cancer could depend on the cell source, cell priming, and culture conditions, as well as on the underlying disease if primary cells are used [38]. We also note that there is not much data on how SC-EVs influence immune cells in cancer. This can probably be explained by the prevailing use of xenograft models to study EV effects in vivo.

Additionally, some initial readouts from the experiments can be interpreted in different ways. For example, decreased proliferation and migration can be seen as a positive effect, but they can also indicate the gain of cancer cell dormancy, that renders cells chemoresistant [92]. In the future, further testing of native EV fractions using more advanced tools, e.g., genetic cancer models or in vitro migration assays that avoid conflating cell motility with proliferation, could be beneficial and reveal more about the action of native SC-derived EVs. Nevertheless, the accumulating data from such experiments have already highlighted several molecules with anti-tumour effects and are instrumental for some of the EV modification approaches that are discussed in the following sections.

## 4. Effects of Modified SC-EVs on Cancer

In this chapter, we will summarise the use of modified SC-EVs in novel cancer treatment strategies (additionally, see Table 1 and Table 2 and Figure 1c,d).

These studies demonstrate that pre- and post-loading of small-molecule drugs, proteins, and small RNA into SC-EVs via different methods can be effective and modified MSC-EVs hold promising potential for cancer treatment applications. However, depending on the approach, some limitations still exist, including poor packaging efficiency, decreased stability, and potential immunogenicity and toxicity [126,127]. Therefore several parameters like the cell source, the yield of EVs, the EV cargo encapsulation method, and the choice of the therapeutic agent are important elements to consider before moving into a clinical setting. Also, modified EV production and isolation protocols should be developed and standardised to produce high-quality clinical grade EVs similar to those described in MISEV2018 [39]. While EV modification protocols depend on the desired application, in the future, the combination of different techniques could improve and even broaden the applications and efficiency of MSC-EVs.

### 4.1. Drug Loading

There are several approaches to load therapeutic agents like chemotherapy drugs into EVs. For example, treating MSCs with Paclitaxel (PTX) promotes MSCs to sort this drug into EVs. These EVs are functionally active in a pancreatic adenocarcinoma cell line, suggesting that MSCs can be used as small factories to produce targeted cancer drugs [101]. The same approach was reported as a potential breast cancer treatment strategy. The authors showed that BM-MSCs treated by PTX produced EVs with enclosed PTX. Administration of these EVs significantly decreased breast cancer cell viability and growth in xenografts [102]. Moreover, Melzer et al. [117] reported that UC-MSCs incubated with taxol produce EVs with the drug enclosed. Treatment with these EVs caused 80–90% cytotoxicity in lung cancer (A549), ovarian cancer (SK-OV-3), and breast cancer (MDA-hyb-1) cell lines compared to MSC-EVs with no taxol, which showed very little cancer cell growth inhibition. Interestingly, taxol-loaded EVs from HUVEC endothelial cells displayed much less effect on the same cancer cell lines compared to loaded MSC-EVs. Systemic intravenous application of MSC-derived taxol-loaded EVs caused more than 60% reduction of primary subcutaneous tumours in vivo, while 50% fewer metastases were observed in distant organs. Similar results were observed with taxol alone, although the concentration of the drug in EVs was about 1000-fold reduced. These strategies are considered as pre-loading techniques, where cargo is loaded into the cells and encapsulated into EVs during their biogenesis. This approach relies on high drug concentrations, long incubation times, and endocytosis of the therapeutic agent. These factors can cause reproducibility challenges.

Another approach is to load drugs directly into EVs after EV isolation, and this technique is called post-loading. One study that systematically compared the cancer cell line MDA-MB-231, the endothelial cell line HUVEC and BM-MSC-derived EV loading with model drugs (porphyrins of different hydrophobicity) by passive co-incubation, electroporation, saponin, extrusion, and dialysis showed that saponin allowed an 11-fold higher drug loading compared to passive methods. The authors also showed that hydrophobic compounds were loaded into EVs significantly more efficiently than into standard liposomes. Loading into EVs increased the cellular uptake of the drug by 60% in vitro compared to a free or liposome-encapsulated drug [128]. However, the main drawbacks of post-loading are that only relatively small molecules can be loaded and that the method has relatively low efficiency and large variability among studies [129,130].

### 4.2. Protein Loading

Considering therapeutic protein post-loading into EVs, several strategies have been described, including sonication and freeze/thaw cycles [131]. However, there are no studies published so far regarding protein post-loading into SC-EVs with these methods in the context of cancer treatment.

One of the most common approaches to pre-load proteins into EVs is to stably transfect SCs by applying a lentivirus system to overproduce certain proteins. A recent study showed that overexpressing TRAIL (TNF-related apoptosis-inducing ligand) in MSCs by a lentivirus system resulted in approximately 95% of EVs containing the protein. Interestingly, TRAIL overexpression also induced EV secretion, suggesting that this protein could be involved in EV biogenesis. These EVs induced apoptosis in 11 cancer cell lines in a dose-dependent manner compared to recombinant TRAIL [123]. Clinical trials using recombinant TRAIL had shown poor benefits in cancer treatment because of limited bioavailability, resistance to this ligand, and low activity [132]. One of the explanations authors provided for the observed differences is that the TRAIL enclosure into EV membranes allows higher clustering of the ligand, which is necessary for effective activation of the extrinsic death pathway [123]. Considering endogenous protein loading into EVs, another sophisticated method called EXPLOR (exosomes for protein loading via optically reversible protein-protein interactions) has been developed [133]. This approach has not been tested in MSC-EVs so far.

### 4.3. RNA Loading

Compared to small molecule drugs, nucleic acid loading into EVs is more challenging due to the size and molecular charge. One of the most often used nucleic acid pre-loading approaches was reported by Munoz and co-authors [103]. They transfected MSCs with anti-miRNA-9-Cy5 oligonucleotide and showed that the majority of anti-miRNA-9 was transferred to the glioblastoma multiforme (GBM) cells by EVs and reversed the expression of multidrug resistance 1 in temozolomide-resistant GBM cells. Likewise, BM-MSC transfection with synthetic double-stranded Cy3-miRNA-124 and Cy3-miRNA-145 resulted in localisation of this miRNA into EVs. Delivery of the miRNA-124 mimic via MSC-EVs decreased migration and self-renewal of glioma cells derived from GBM specimens [104]. Similarly, miRNA-124 transfected WJ-MSCs produced EVs with more miRNA-124. Delivery of miRNA-124 to GBM cells enhanced their sensitivity to temozolomide and decreased migration in vitro, suggesting that miRNA delivery by WJ-MSC-derived EVs could provide a new strategy for miRNA replacement therapy in GBM cancers [118]. Likewise, BM-MSC transfection with synthetic double-stranded miRNA-143 resulted in an increased number of EVs containing miRNA-143 that could inhibit the migration of the osteosarcoma cell line 143B compared to control EVs [105].

A slightly different approach was applied by transfecting MSCs with miRNA-146b expression vector by electroporation. This resulted in a 7.3 fold increased miRNA-146b concentration in MSC-secreted EVs compared to controls. Intratumour injection of these EVs significantly reduced glioma xenograft growth in a rat model of primary brain tumour compared to control EVs, suggesting that sorting of therapeutic miRNA into MSC-EVs represents a new potential treatment strategy for malignant glioma [106]. The same approach was reported by Lou and colleagues [114] who transfected AD-MSCs with miRNA-122 expression vector producing EVs with an increased amount of miRNA-122. Intratumour administration of these EVs by injection significantly improved the anti-tumour efficacy of sorafenib in a hepatocellular carcinoma (HCC) model in vivo. Another study demonstrated that dormant breast cancer cells prime MSCs in vitro to secrete EVs with miRNA-222/223, which can induce cellular quiescence and drug resistance in some types of cancer cells. According to these findings, dormant breast cancer cells could be targeted by the systemic application of MSCs transfected with anti-miRNA-222/223 resulting in increased sensitivity to chemotherapy and an increased survival rate in vivo [107]. However, Ma et al. demonstrated the opposite effect of miRNA-221 carrying EVs produced by BM-MSCs transfected with a miRNA mimic. These EVs increased proliferation, migration, invasion, and adhesion of the gastric cancer cell lines BGC-823 and SGC-7901 [95].

Similar to proteins, also overexpression of RNA by lentivirus system results in their increased EV enclosure. For example, O’Brien and colleagues engineered adult BM-MSCs with lentivirus to secrete EVs enriched with tumour suppressor miRNA-379 [109]. The authors demonstrated that systemic administration of cell-free EVs enriched with miRNA-379 had a therapeutic effect in vivo through regulation of COX-2 (cyclooxygenase-2), while the administration of miRNA-379 engineered BM-MSCs resulted in no adverse effects. Moreover, the addition of BM-MSC-EVs containing miRNA-124a to glioblastoma stem cells (GSC) caused a significant reduction in cell viability and clonogenicity. In vivo treatment resulted in 50% of animals living long term, and histological analysis of the survivors did not show the presence of tumours [110]. Another possible approach to load RNA into SC-EVs is to manipulate endogenous RNA sorting mechanisms. There is evidence that the RNA profile in EVs depends on the type and origin of the parental cells, and some RNAs are enriched in certain cell types [134]. These observations suggest that distinct mechanisms exist for RNA sorting into EVs, and these mechanisms could be used to deliver specific therapeutic RNAs to EVs in a more sophisticated manner by applying gene engineering. Currently, there are several known possible RNA sorting mechanisms involving the RISC (RNA-induced silencing complex), the ceramide pathway, miRNA-mRNA ratio, non-template terminal nucleotide additions and ribonucleoprotein interaction with a specific RNA sorting sequence motif [135]. Commercially available systems like the XMIRXpress lentivirus system (SBI System Biosciences) that contains a miRNA sorting sequence motif or an artificially introduced RNA sorting sequence could be applied to MSCs in the future.

RNA post-loading approaches have also been studied in SC-EVs. For example, Greco et al. used electroporation to load PLK1 (polo-like kinase 1) siRNA into EVs derived from HEK293 [122]. The subsequent co-incubation of these EVs with bladder cancer UMUC3 cells increased their PLK1 siRNA concentration by almost 30 times. Similar results were obtained with MSC-derived EVs. Eventual co-incubation of UMUC3 cells and MSC-EVs loaded with siRNA showed that suppression of PLK-1 induced both apoptosis and necrosis in UMUC3 cells. Similarly, BM-MSC-EVs were efficiently loaded with LNA (locked nucleic acid) anti-miRNA-142-3p by electroporation and successfully used to reduce miRNA-142-3p levels and restore *Apc* and *P2x7r* gene expression in mouse breast cancer cell lines (4T1 and TUBO) in vivo [108].

### 4.4. SC-EV Modifications to Improve Tissue Targeting

While there are several studies done to modify SC-EV content, additional research needs to be performed to modify the membrane of SC-EVs with a technology called EV display [126,136,137,138]. While systemically administered EVs can accumulate in undesired locations [138], EV display technology might allow engineering EV membranes in a way that EV uptake in target tissues is increased. So far, several approaches have been developed like overexpression of specific EV membrane proteins, antibody/antigen conjugation, modification of surface proteins, EV surface synthetic modification, or hybrid EV production. For example, Sato et al. proposed a new technique for tailoring EVs with desired characteristics based on the direct membrane fusion between pre-isolated EVs and synthetic liposomes [139]. This approach enables to obtain personalised EVs where multiple ligands can be inserted into a variety of preformed liposomes containing a number of drugs. Another option is to use commercial products like XStamp (SBI System Bioscience), that allow virtually unlimited targeting options by decorating EVs with streptavidin, followed by binding of biotinylated targeting molecule. However, to the best of our knowledge, there are no reports of EV display technology in MSCs for cancer treatment.

### 4.5. Increase of SC-EV Yield

Besides MSC-EV modification for loading and targeting purposes, some studies aim to increase MSC-EV yield with engineering methods. The rationale behind this is that finite MSC expansion capability for EV generation, and the yield of MSC-EVs are limiting factors in large scale production for cell-free therapies. There are some good manufacturing practice (GMP)-grade standard protocols for MSC-EV isolation [140], but they suffer from the same problem. One of the solutions is to immortalise MSCs. For example, Lai and colleagues demonstrated that the production of EVs is scalable under stringent GMP conditions using MSCs immortalised by overexpression of c-Myc [141]. An alternative cell source for EV production are PSC-derived MSC-like cells, that can be robustly induced in vitro (iMSCs—induced MSC-like cells). Current in vitro studies indicate that iMSCs could overcome EV production limitations. However, more studies are needed to demonstrate the safety of such an approach [142]. Besides engineering, other methods to increase EV yields like lowering pH in the culture medium, hypoxia treatment, small molecule treatment, 3D ECM, large scale expansion methods, i.e., spinner flasks and hollow-fibre bioreactor among other methods, have been tested in MSCs [137,143,144]. The current achievements of methods to increase the production of MSC-derived EVs are reviewed by Wang et al. [143] and Phan et al. [144].

## 5. Adult SCs versus EVs: Advantages and Disadvantages in Cancer Treatment

In the last decades, SCs have raised increasing interest regarding their therapeutic potential not only in regenerative medicine but also in cancer treatment. SCs have the intrinsic ability to migrate towards inflammatory and tumour sites and exert anti-tumour and immunomodulatory activities [145,146,147]. Although the mechanism of MSC tropism is not fully understood, several studies indicate that it may depend on different chemoattractant-receptor pairs, such as the stromal cell derived factor 1 and its receptor C-X-C chemokine receptor type 4, or monocyte chemoattractant protein-1 and C-C chemokine receptor type 2 receptor [148]. Moreover, genetically engineered SCs that express higher levels of chemokine receptors are more efficient in targeting glioma cells [149], thus providing a powerful tool to increase homing and improve treatment outcomes.

SCs naturally secrete therapeutic substances that inhibit cancer development. Friedman et al. [150] reported that human UC-MSCs secrete significant amounts of cytokines, including TGF-β, tumour necrosis factor-alpha, and interferon alpha and gamma. These molecules have both direct anti-proliferative and pro-apoptotic effects and an immunostimulatory effect that potentiates the anti-cancer immune response, resulting in a dual attack. Moreover, native MSCs have been described to release Dickkopf related protein 1, leading to the inhibition of Wnt signaling pathways, which ultimately affects tumour cell cycle [151,152]. Nevertheless, various studies have also reported a pro-cancer behaviour of adult SCs [153]. In a xenograft model, the mixing of human colorectal cancer cells with MSCs resulted in increased angiogenesis and tumour growth rate [154]. The authors claimed that the secretion of IL-6 from MSCs, which in turn induces endothelin-1 release from cancer cells and the following recruitment of endothelial cells upon activation of Akt and ERK, is responsible for the observed enhanced angiogenic capacity. These opposite pro- and anti-tumour effects of SCs could possibly be explained in the light of the source of SCs. In fact, SCs have been demonstrated to be sensitive to microenvironment priming, i.e., MSCs derived from neoplastic pancreas promote cancer growth to a larger extent than the healthy counterpart [155]. Moreover, Sheng et al. showed that proinflammatory cytokines, such as IFNγ, play an important role in priming the immunosuppressive property of MSCs [156].

Despite the large number of preclinical studies that use SCs for cancer therapy, their application in cancer treatment clinical trials is still relatively rare compared to other clinical conditions. One of the main reasons is the safety concerns regarding SC transplantations in patients. Although less immunogenic than other allogeneic cells, allogeneic MSCs have been shown to elicit an immune response in vivo resulting in their rejection [157], thus meaning that they should not be considered as immune-privileged but rather to have the ability to escape host rejection transiently. A possible way to overcome host rejection is to apply autologous SCs, either directly derived from patients or reprogrammed from adult somatic cell iPSCs. Both approaches have limitations since patient-derived SCs could be already primed towards a pro-tumourigenic behaviour [155] and iPSCs, which are forced to express pluripotency factors, tend to form teratomas in mice [158,159]. A possible strategy to prevent SC neoplastic transformation in vivo is to engineer them in order to express a “suicide gene” that converts non-toxic prodrugs into cytotoxic products [160]. Once transplanted into tumour-bearing models, engineered SCs localise to tumour tissues, and the exogenous enzyme converts systemically administrated prodrug into a cytotoxic molecule. As a result, a high concentration of the cytotoxic drug is locally acquired, which harms tumour cells with no or few off-target effects and eradicates SCs at the same time. Cultivation of SCs in vitro introduces a bias that could potentially impact the therapeutic outcome. In particular, long-term cultures of MSCs have been shown to undergo spontaneous malignant transformation [161]. Senescence bypass upon deletion at the Ink4a/Arf locus and hyperphosphorylation of retinoblastoma protein are suggested as an explanation [162]. Also, the in vivo therapeutic effects of MSCs are hampered by the relatively large size of the cells (15–30 µm in diameter) that leads to mechanical entrapment of MSCs in lung capillaries within minutes after intravenous injection. The cells are then redistributed to the liver and spleen and finally cleared by phagocytes within 7–10 days after their administration [163]. This indicates that the biodistribution of exogenous MSCs is rather limited to lung, liver, and spleen and has reduced accessibility to other target organs. Indeed, only 0.2% of MSCs were detected in a subcutaneous xenograft of PC3 cells in a prostate cancer model at day seven post-infusion [164]. Ultimately, these safety concerns reduce the clinical application of SCs for cancer therapy and underline the need to move to more easily standardisable cell-free systems.

To improve the tumour-targeted delivery and therapeutic efficacy of MSCs, it has been suggested to either use EVs secreted by native or engineered MSCs or to fabricate synthetic MSC therapeutic particles that possess the paracrine activity of the parent MSCs and escape lung entrapment [164,165,166].

Previous studies have revealed that MSC-EVs participate in the paracrine transfer of signalling molecules and, similarly to MSCs, could regulate tumour cell proliferation, angiogenesis, and metastasis [167]. SC-EVs share many similarities with their donor cells, including similar immunophenotype, protein signature, RNA content, and functional properties that make them an attractive model for novel targeted cell-free anti-cancer therapy design. Comparing to adult SCs, SC-EVs have several advantages as therapeutic agents (see Table 3). 

For example, as other EVs, they can pass biological barriers, like the BBB, blood-cancer barrier, and shuttle bioactive molecules from one cell to another, also causing the exchange of genetic information and reprogramming recipient cells. Moreover, SC-EVs have been considered as an attractive therapeutic agent shuttle to cancer cells thanks to their cancer cell tropism, which is similar to their parental cells, and to a more efficient internalisation in cancer cells than liposomes of comparable size [168]. Adult SC-EVs also have prolonged time in the circulation due to the smaller size, and they are not trapped to the same extent in the lungs, liver, or spleen compared to MSCs. SC-EVs raise fewer safety concerns as they are not capable of undesired proliferation and differentiation upon administration in the body. 

Additionally, adult SC-EV tissue targeting efficiency can be enhanced by surface protein engineering and the versatility of cargo molecules that can be uploaded by passive or active methods. SC-EVs are also easier to handle, less expensive, and can be generated on a relatively large scale [87]. However, there are several challenges to overcome to make SC-EVs a superior cancer treatment (See Table 3). There is no standardisation in the usage of SC-EVs in vitro and in vivo studies, which could be one of the main factors limiting the start of new clinical trials. The lack of consistency between studies makes it difficult to compare existing studies. Therefore, the reproducibility of EV purification and characterisation methods have to be established. There are already established GMP protocols for MSC-EV isolation, as mentioned in a previous chapter [140]. Moreover, there is no standard way to quantify the number of EVs given to cells. EV concentration measurements are often based on EV protein concentration, which varies significantly between studies, and the number of particles per milliliter is not considered a precise method of EV quantification [169]. Thorough preclinical pharmacokinetics and pharmacodynamics evaluation have to be done before EV-based anti-cancer therapeutics can be used in the clinic. Several initiatives are taken to functionally test and characterise MSCs to determine their potency before application, and SC-EV production could benefit from adopting similar procedures [170]. In summary, there are clear advantages of EVs over SCs. Therefore MSC-EVs are promising therapeutic agents for cancer treatment in the future. Current MSC-EV pre-clinical application results in vivo demonstrate almost no side effects compared to cellular therapies [171]. Also, modified or engineered SC-EVs might prove to be more effective than native MSC-EV in the future, but more studies and standardised protocol for modified EV production and characterisation are necessary.

## 6. Perspectives and Future Challenges of Adult SC-EV in Cancer Treatment

Currently (April 2020), there are over 160 trials listed on www.clinicaltrials.gov involving exosomes, 47 trails using EVs, 298 trials with MVs and/or microparticles, and 31 mentioning the use of apoptotic bodies. While 83 studies use exosomes in cancer clinical trials, nine cancer trials involve EVs, 56 cancer trials involve MVs or microparticles, and 18 cancer trials mention apoptotic bodies. Only 13 trials use the term SC exosomes, five SC-EVs, 33 involve SC-MVs or microparticles, and three mention SC apoptotic bodies.

So far, there are only two clinical trials that use AD-MSCs and fibroblast-like MSCs (FL-MSCs) as an EV source for cancer treatment. The study entitled “iExosomes in Treating Participants With Metastatic Pancreas Cancer With Kras^G12D^ Mutation” (NCT03608631) plans to use FL-MSC-EVs engineered to carry siRNA or shRNA specific to oncogenic KRAS^G12D^ to treat pancreatic ductal adenocarcinoma. In the *in vivo* proof-of-concept study, iExosomes containing KRAS^G12D^ siRNA induced sustainable tumour growth inhibition in an orthotropic mouse model [172].

The trial “The EXOPRO Study: How Does Prostate Cancer Metastasize?” aims to explore the role of EV communication between patient-derived adipose tissues, presumably including also adult SCs and prostate cancer cells lines to find out the differences in prostate cancer progression between lean and obese patients (NCT04167722). The effect of small RNAs transferred by human adipose tissue EVs on prostate cancer regulation will be analysed. The study started recruiting patients in 2019, and the estimated completion date is 2023. Since cancer treatment clinical trials using SC-EVs are generally novel, it is premature to make any conclusions.

To promote SC-EV application in cancer treatment, there are still fundamental and methodological questions that should be addressed to fully understand the biology of these EVs. As we have discussed previously, SC-EVs clearly have the ability to influence various aspects of tumour progression. Mixed outcomes are observed using native EVs, while more unified responses are reported when EVs are modified. In addition, there is a clear need to do more studies involving in vivo models or sophisticated in vitro systems, like organoid co-cultures or organ on a chip, to validate the effects of SC-EV on tumourigenesis before starting cancer clinical trials and subsequent therapy.

Despite the established immunomodulatory action of MSCs [173] and EVs [174,175] and the importance of EV-related immune evasion response in tumour progression [176], we could not find many studies demonstrating direct SC-EV-mediated immune modulation. Perhaps this could be addressed by using genetic tumour models as recently demonstrated [177], or more advanced in vitro platforms. For example, tumour organoid-T-cell co-cultures models are available to study tumour-immune cell interactions and could be adapted to include stem and progenitor cells or EVs [178]. The use of such models could also help to address the importance of cells versus EVs for immunomodulatory response in future cancer therapies.

The realisation that transplanted MSCs are short-lived and that the effects of infused cells could be mediated by their secretome has produced the concept of a cell-free MSC-based therapy that would include EVs and other components released by cells [30]. A recent report has questioned the need for a living fit MSC in a transplantation product and suggested that apoptotic cells could be used for immune modulation [179]. Along these lines, another report showed that MSCs are phagocytosed by the monocytes that subsequently modulate the adaptive immune system [180]. It would be interesting to uncover if these observations are related to the apoptotic bodies released by MSCs, whether EVs can be phagocytosed to trigger a similar immune modulation and what implications it might have in cancer models. Some data already suggest that stressed MSCs [71,91] and some apoptotic cells [181] do release EVs that can modulate macrophages and attenuate inflammation.

Another intriguing aspect is the role of endogenous progenitor- and SC-derived EVs in cancer. Based on the involvement of EVs in developmental signaling [85,182], one could assume that some of the functions of adult SCs in tissue repair and cellular plasticity could also be EV-mediated. Given the many parallels between mechanisms seen in tissue repair and cancer [83], further speculation can be made about the possible involvement of endogenous adult SC-EVs in cancer progression. Transplanted MSCs are known to display injury and tumour tropism [15], and perhaps endogenous adult SCs perceive tumour lesions in a similar way and engage in EV-mediated regulation. What makes this path particularly challenging is identifying the origin of EVs. A better understanding of EV subpopulations, unique marker identification, and being able to label and track the EVs from their origin to acceptor cell [183] and between organs [184] could greatly facilitate this research area in the future. Alternatively, some insights can be gained from in vitro models. Recent advancements in organoid culture methods have allowed to expand adult SCs and progenitor cells from different organs and study them in settings that better resemble the in vivo environment [185].

In conclusion, SC-EVs demonstrate a high potential for cancer therapy. Moreover, engineered SC-EVs are anticipated to find applications in a unique niche between molecular and cellular medicine and play a part in personalised cancer therapy. However, our fundamental knowledge of EV biology is in a relatively early stage, and much effort needs to be made in order to guarantee their safe and effective therapeutic use. Moreover, adequate standards for SC-EV manipulation need to be established to bring SC-EVs a step closer to clinical application.

## Figures and Tables

**Table 1:** Summary of pro-cancer effects of MSC-EVs.

EV Source	Target	Cargo/Mechanism	Method	Effect
BM-MSC				
Human primary	GC, colon cancer cells	↑VEGF through ERK1/2	In vivo	↑growth & angiogenesis [89]
MM patients primary	multiple myeloma cells	miRNA15a, oncogenic proteins	In vivo	↑homing to BM & growth [38]
Human primary	breast cancer cells	miRNA21, miRNA34a	In vivo	↑growth & angiogenesis [37]
Human primary	osteosarcoma, GC cells	HH pathway activation	In vitro	↑growth & migration [90]
Human commercial	lung cancer cells A549 and H23	hypoxia-induced miRNA-21-5p	In vitro In vivo	↑proliferation, survival, invasiveness, EMT ↓apoptosis, macrophage M2 polarisation [91]
Human commercial	metastatic breast cancer cells	suppression of *MARCKS* by miRNA23b	In vivo, clinical sample analysis	↑cancer cell dormancy and ↓sensitivity to docetaxel [92]
Human primary	nasopharyngeal carcinoma cells	FGF19 activated FGFR4-dependent ERK cascade	In vivo	↑EMT, ↑tumour incidence & growth [93]
p53-/- knockout mouse primary	p53+/+ primary mouse BM-MSC and MFC cells	UBR2 protein and mRNA	In vitro	↑proliferation, migration, expression of stemness related genes [94]
Human primary	GC cells	miRNA-221 pre-loaded	In vitro	↑proliferation, migration, invasion, and adhesion [95]
AD-MSC				
Human primary	breast cancer cells	Wnt pathway activation	In vitro	↑ migration [96]
UC-MSC				
Human primary	GC cells	↑ CaMKs -Raf/MEK/ERK pathway	In vivo	↑ resistance to 5-fluorouracil ↓apoptosis [97]
Human primary	lung adenocarcinoma cells	miRNA-410 transfer, ↓PTEN expression	In vivo, in silico prediction	↑growth, ↓apoptosis [98]
Human primary	breast & ovarian cancer cells	enzyme transfer	In vitro	↑ cancer cell heterogeneity [99]
Wharton’s jelly primary	renal cancer cells	↑HGF, ↑ ERK1/2 and AKT pathways	In vivo	↑ tumourigenesis & tumour growth [100]

**Table 2:** Summary of anti-cancer effects of native, modified, and engineered adult SC EVs.

EV Source	Target	Cargo/Mechanism/ Modification	Method	Effect/Reference
BM-MSC				
Human commercial	Kaposi sarcoma, ovarian cancer, hepatoma cells	N/A	In vivo	↓growth, ↑apoptosis [50]
Mouse commercial	breast cancer cells	miRNA-16, ↓VEGF	In vivo	↓growth & angiogenesis [36]
Human primary	multiple myeloma cells	lack of miRNA-15a & oncogenic protein transfer	In vivo	↓homing to BM & growth [38]
Mouse commercial	human pancreatic cells CFPAC-1	PTX pre-loaded EVs	In vitro	↓proliferation [101]
Human commercial	breast cancer cells MDA-MB-231	PTX pre-loaded EMs	In vitroIn vivo	↓viability ↓tumour growth [102]
Human primary	human commercial and primary GBM cells	Cy5-tagged anti-miRNA-9 pre-loaded EVs	In vitro	↓chemoresistance to TMZ [103]
Human commercial	human-derived glioma cells and GSC	Cy3-miRNA-124a & miRNA-145 pre-loaded EVs	In vitro In vivo	↓migration of glioma cells and the self-renewal of GSCs [104]
Human commercial	human osteosarcoma cells 143B	miRNA-143 pre-loaded EVs	In vitro	↓migration [105]
Rat primary	rat model of primary brain tumour	miRNA-146b pre-loaded EVs	In vivo	↓glioma xenograft growth [106]
Human primary	Breast cancer cells MDA-MB-231 and T47D	Anti-miRNA-222/223 pre-loaded EVs	In vivo	↑carboplatin-based therapy efficiency [107]
Mouse primary	mouse breast cancer cells 4T1 and TUBO	LNA-anti-miRNA-142-3p post-loaded EVs	In vitro In vivo	↑APC and P2X7R expression [108]
Primary human	T47D and HCC-1954 (HCC) breast cancer cells	miRNA-379 pre-loaded EVs	In vitroIn vivo	↓COX-2 ↓tumour formation and growth rate [109]
Human commercial	5 GSC primary cells	miRNA-124a pre-loaded EVs	In vitro In vivo	↓ FOXA2, viability, clonogenicity, ↑survival [110]
AD-MSC				
Human commercial	metastatic prostate cancer cells	miRNA-145	In vitro	↓growth, ↑apoptosis [111]
Human primary	ovarian cancer cells	miRNA mediated ↓BCL2	In vitro	↓growth & migration, ↑apoptosis [112]
Rat primary	Hepatocellular carcinoma animal model	NKT-cell anti-tumour response	In vivo	Improved tumour grading, ↑NKT-cells [113]
Human commercial	human-derived glioma cells and GSC	Cy3-miRNA-124a & miRNA-145 pre-loaded EVs	In vitro In vivo	↓migration of glioma cells and the self-renewal of GSCs [104]
Human primary	human liver cancer cell line HepG2	miRNA-122 pre-loaded EVs	In vitro In vivo	↑anti-tumour efficacy of sorafenib [114]
UC-MSC				
Human primary	Breast cancer lines	miRNA-148b-3p by regulating TRIM59 expression	In vivo	↓EMT, tumour growth, ↑apoptosis [115]
Wharton’s jelly primary	bladder carcinoma	inhibition of Akt pathway, cleaved Caspase3 induction	In vivo	↓growth, ↑apoptosis [116]
Human primary	cancer cell lines: A549 SK-OV-3 MDA-hyb1	Taxol pre-loaded EVs	In vitro In vivo	↑cytotoxicity ↓subcutaneous primary tumours & metastasis [117]
Human commercial	human-derived glioma cells and GSC	Cy3-miRNA-124a & miRNA-145 pre-loaded EVs	In vitro In vivo	↓migration of glioma cells and the self-renewal of GSCs [104]
Wharton’s jelly primary	GBM cells U87	miRNA-124 pre-loaded EVs	In vitro	↓CDK6 ↑Chemosensitivity to temozolomide [118]
Other ASCs				
Human liver MSCs primary/ commercial	Hepatoma [119], tumour-derived endothelial cells [120], renal CSC [121]	miRNA-31, 451 [119] miRNA-15a, 181b, 320c, 874 [120] miRNA-145, 200 [121]	In vivo	↓tumour growth [119] ↓angiogenesis [120,121], ↑apoptosis [121] delayed metastasis [121]
Human placenta-derived MSC commercial	human-derived glioma cells and GSC	Cy3-miRNA-124a & miRNA-145 pre-loaded EVs	In vitro In vivo	↓migration of glioma cells and the self-renewal of GSCs [104]
MSC with unspecified origin	bladder cancer cells UMUC3 & SW780	PLK-1 siRNA post-loaded EVs	In vitro	↓PLK-1 expression ↑apoptosis and necrosis [122]
Human MSC with an unspecified origin	11 different cancer cells	TRAIL pre-loaded EVs	In vitro	↑apoptosis in 11 cancer cell lines including TRAIL-resistant cells [123]
Human MenSC primary	chemically-induced OSCC	N/A	In vivo	↓ tumour growth, ↓angiogenesis [124]

**Table 3:** Advantages and disadvantages of MSC-EVs over MSCs.

Advantages	Disadvantages
1. Less mechanical entrapment in tissues 2. Paracrine function 3. Tumour homing similar to MSCs 4. Good safety profile 5. Modification options 6. Easier and less costly to handle 7. Yield can be easier increased compared to MSCs	1. Lack of standardised production, modification, and characterisation 2. Modifications can cause immunogenicity or toxicity risk 3. Potential of tumour promoting effects similar to MSCs 4. Lack of information on tumour selectivity depending on the EV source
